# Small mosquitoes: large implications—effects of larval crowding and starvation on locomotor activity, adult biting frequency, and insecticide resistance in two strains of *Aedes aegypti*

**DOI:** 10.1186/s13071-025-06886-w

**Published:** 2025-07-01

**Authors:** Anjali Karki, Hailey A. Luker, Keyla R. Salas, Immo A. Hansen

**Affiliations:** Department of Biology, New Mexico State University, Las Cruces, NM USA

**Keywords:** *Aedes aegypti*, Larval crowding, Starvation, Locomotor activity, Adult biting, Insecticide resistance

## Abstract

**Background:**

Stress during the larval phase of their post-embryonic development can result in reduced-size imagoes in mosquitoes. Water temperature, salinity, food availability, crowding, and predation are factors that affect larval development timing and adult size. In an earlier study we compared the transcriptomes and metabolomes of adult mosquitoes that were raised under standard conditions (large) with mosquitoes raised under stress conditions (small) and found significant changes. Continuing this line of inquiry, we compared the general activity, biting frequency, and insecticide resistance in small and large *Aedes aegypti*.

**Methods:**

In the study, we generated different-sized mosquitoes using larval crowding and nutritional stress. To compare the size-based variation in activity, we used the Locomotor Flight Activity Monitor (LAM-25) and a feeding assay to record the biting behavior of female mosquitoes. We then used a modified bottle assay to assess the levels of insecticide resistance in small and large mosquitoes of different strains.

**Results:**

We found that small and large mosquitoes have different activity and biting patterns over a 2-week time course; however, the cumulative number of engorgements was not different. After pyrethroid exposure, knockdown curves of small and large mosquitoes were similar in the susceptible University of Georgia Laboratory (UGAL )strain but different in the insecticide-resistant Puerto Rico strain.

**Conclusions:**

Our results highlight the large knowledge gaps regarding the effects of mosquito size on vectorial capacity.

**Graphical Abstract:**

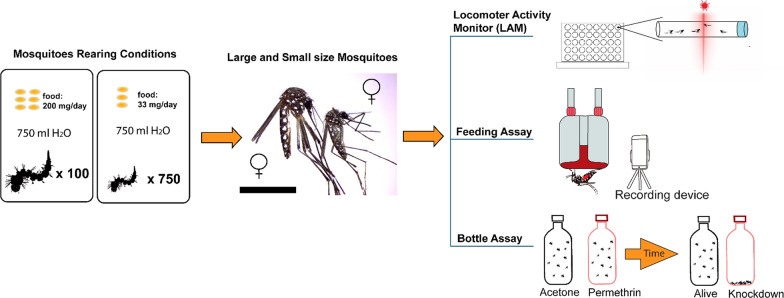

**Supplementary Information:**

The online version contains supplementary material available at 10.1186/s13071-025-06886-w.

## Background

Size variations in mosquitoes and other multicellular organisms are caused by genetic factors and environmental circumstances during post-embryonic development [1, 2]. The underlying mechanisms involved in the determination of the size of an organism have been studied extensively at the cellular, tissue, organ, and organism levels. Molecular mechanisms that can influence body size in insects include insulin signaling, amino acid signaling, and the ecdysone signaling pathway [3, 4]. Intriguingly, these molecular pathways are the same ones that regulate insect reproduction.

Specific environmental factors known to impact insect size during developmental stages include temperature, nutrition, population density, and predation. Environmental temperature and insect body size are typically negatively correlated; warmer conditions result in smaller body sizes, and cooler conditions often result in larger body sizes [5–7]. The availability of nutrients is strongly and positively correlated with insect body size, while high population densities are negatively correlated. Factors such as predation have also been shown to negatively affect size in mosquitoes [8].

Mosquito imagoes of different sizes can be easily produced in laboratory environments. The manipulation of available nutrition and/or crowding during the larval phase is used to produce smaller or larger mosquitoes [9]. The size of the first instar larva is determined by the size of the egg, while the size of the subsequent instars can vary significantly on the basis of rearing conditions [10]. It has been recognized for some time now that a mosquito’s size affects their reproductive physiology. There are several studies exploring this topic using the yellow fever mosquito, *Aedes aegypti*, as a model. In a study from 1980, Feinsod and Spielman showed that small *Ae. aegypti* females that were nutrient-deprived during the larval phase required a blood or sugar meal for their ovaries to become reproductively mature. The topical application of juvenile hormone (JH) had the same effect on the maturation of ovaries in small mosquitoes [11]. Studies from 2003 and 2004 showed that smaller female *Ae. aegypti* had lower levels of juvenile hormone (JH) in their hemolymph [12, 13]. In 2015, Price and coworkers identified differentially expressed genes in the fat bodies of large and small mosquitoes [9]. Many of these genes were identified as immune genes. These findings suggest a correlation between mosquito size and vectorial capacity. The vectorial capacity of a mosquito vector is the likelihood of pathogen transmission by the vector to a susceptible population. It is calculated using five parameters: female mosquitoes per human, biting rate, vector competence, probability of mosquito survival, and the extrinsic incubation period [14, 15].

We hypothesized that mosquito size impacts most of the parameters used to calculate vectorial capacity. In the following study, we investigated the effects of mosquito size on locomotor activity, biting frequency, and insecticide resistance using the yellow fever mosquito, *Aedes aegypti*.

## Methods

### Mosquito strains

The *Aedes aegypti* mosquito strains University of Georgia Laboratory (UGAL) and Puerto Rico were reared as described in Marquardt (2004) [16]. Puerto Rico eggs were generously provided by Alden S Estep III (US Department of Agraculture [USDA], Agricultural Research Service [ARS], Southeast Area Mosquito & Fly Research Unit, Gainesville, FL). This strain is homozygous for the F1534C mutation in its voltage-gated sodium channel and is highly resistant to pyrethroids [17].

### Generation of large and small mosquitoes

**Fig. 1:**
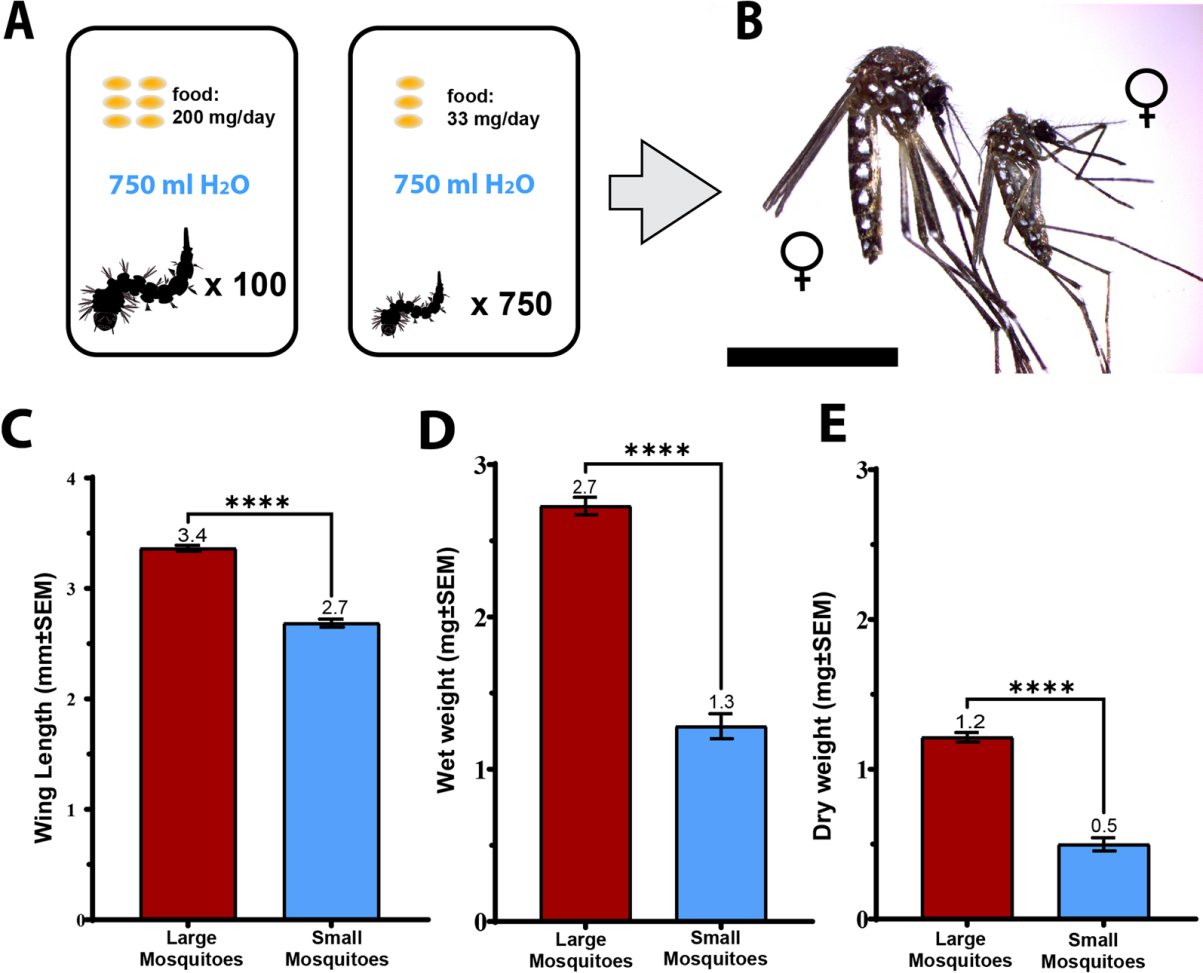
Body size and weight of large and small *Aedes aegypti*. **A** Schematic of rearing conditions for the production of different-sized mosquitoes. **B** Large (left) and small (right) *Ae. aegypti* females. Scale bar depicts 5 mm. The average wing length (**C**), wet weight (**D**), and dry weight (**E**) of large and small mosquitoes. Unpaired *t*-tests were performed for comparisons. ^****^P < 0.0001

Larval crowding and nutritional stress were used to generate different-sized mosquitoes from the same batch of eggs as described in Shiao et al. [46] and Price et al. [9] with minor modifications. Mosquito eggs were hatched in pans (32 × 23 × 5 cm) filled with 750 mL de-ionized water with an added cat food pellet. Then, 2 days after hatching, specific numbers of second-instar larvae were distributed into different pans using the tally counter and transfer pipette. To generate large mosquitoes, groups of 100 larvae were placed into individual pans. These larvae were fed on 0.2 g of ground Special Kitty cat food (Walmart, Bentonville, AR) every other day. To generate small mosquitoes, groups of 750 larvae were placed into individual pans. These larvae were fed on 0.033 g of ground Special Kitty cat food every other day (Fig. 1A). Pupae were collected from each pan for up to 5 days or until there were no more larvae in the pans. These pupae were placed in BugDorm cages (27.5 × 29.5 × 29.5 cm; BugDorm Company, Taichung, Taiwan) and provided with 20% sucrose solution ad libitum. Adult mosquitoes were reared under standard conditions of a 14:10 light–dark cycle at 27 ℃ and 85% relative humidity. Adult mosquitoes were at least 5 days old before being used in all experiments.

### Weight and wing measurements

Groups of 50 adult mosquitoes were frozen at −80 °C overnight and weighed to record wet weight. Mosquitoes were then dried at 50 °C overnight and weighed again to record dry weight. For the wing length measurement, other groups of 50 adult mosquitoes were used. The wing measurements were recorded using a microscope slide with a millimeter scale and a stereo microscope.

### Locomotor activity patterns

We recorded locomotor activity patterns of adult mosquitoes by using the Locomotor Activity Monitor (LAM) (LAM-25, TriKinetics, USA). In this device, groups of five mosquitoes were kept in 32 horizontal plastic cylindrical tubes (23 mm diameter × 94 mm long) for 24 h. This device was used to measure mosquito activity by recording the number of crossings through infrared beams. Average activity was recorded over 60-min intervals. Five days post-emergence, mosquitoes were aspirated from their respective cages and ice anesthetized for 10 s. Separate groups of five male or five female mosquitoes were then placed in each glass tube (23 mm diameter × 94 mm long) using forceps. For each treatment and sex, 16 replicates were performed. A wet cotton plug was then inserted into the open end of the tubes to a depth of 10 mm, leaving an 84 mm distance for the mosquitoes to move back and forth. After allowing 30 min for the mosquitoes to recover from anesthesia, the tubes were placed in the LAM, where activity was continuously monitored for 24 h.

### Feeding frequency assay

A feeding assay was used to record the feeding behavior of female mosquitoes [18]. For this assay, groups of 25 female mosquitoes were transferred into small BugDorm cages (13 × 13 × 13 cm) (BugDorm Company). A Hemotek membrane feeding system (Hemotek Ltd., Blackburn, UK) containing 1 mL of defibrinated bovine blood (HemoStat Laboratories, Dixon, CA) was placed on the mesh top of the cage as shown in Fig. 3A. Female mosquitoes were offered a blood meal in the morning for 30 min once a day for 14 days. During this assay, female mosquitoes were provided sugar ad libitum. An iPhone 4 (Apple Inc., Cupertino, CA) was used to record the feeding activity during the 30-min duration each day. After the blood meal, mosquito cages were transferred and maintained under standard conditions at 27 ± 2 ℃ with 80% relative humidity under a 12-h light–dark cycle in the mosquito insectary, and egg cups were added to oviposit 2 days following the blood meal. The recordings were reviewed, and the number of engorged females was determined visually for each feeding trial (Supplementary Videos 1, 2). Extension of the abdomen due to blood ingestion was used as a criteria to score a successful bite.

### Insecticide resistance bottle assay

A modified bottle assay was used to detect insecticide resistance phenotypes [19]. Wheaton bottles were prepared 24 h before experiments and stored in a dark area. The control bottles were treated with 1 mL of acetone. Test bottles were treated with 1 mL of acetone containing 86 µg of permethrin (Catalog # 45614, CAS # 52645-53-1 Sigma-Aldrich, St. Louis, MO). For each replicate, 10–15 unfed 5-day-old female mosquitoes were ice anesthetized and added to either treatment or control bottles. Mosquito death over time was determined by counting the number of knocked-down mosquitoes in 5-min intervals. Mosquitoes were considered knocked down if they were unable to upright themselves and were stuck on their backs. Mosquito death was monitored for up to 50 min.

### Statistical analysis

Body weight, wing length, and locomotor activity data were tested for normal distribution using the Shapiro–Wilk normality test. As the data were not normally distributed, we evaluated the statistical significance using the Mann–Whitney *U* test. Feeding frequency data were tested for normal distribution using a Shapiro–Wilk test. As the data were normally distributed, we evaluated the statistical significance of the cumulative engorgements using a paired *t*-test and engorgement per female using a two-way analysis of variance (ANOVA) followed by Tukey’s post hoc test for multiple comparisons. Bottle assay data were analyzed using Fisher’s exact test performed on Python to identify significant differences between the knockdown curves of large and small mosquitoes. All other statistical tests were performed using GraphPad Prism 9 (GraphPad Software, San Diego, CA) with a significance level of α (0.05). The raw data for this study is available in Supplementary File 1.

## Results

### Larval nutrition and uncrowded conditions positively affect the adult size of mosquitoes

We used larval crowding and nutrient availability to generate two groups of adult mosquitoes (Fig. 1A). Standard rearing of larvae in nutrient-rich and uncrowded conditions produced “large mosquitoes.” “Small mosquitoes” were produced by rearing larvae in nutrient-deprived and overcrowded conditions (Fig. 1B). We measured the wing length and body weight of adult mosquitoes to differentiate between these size groups. The average wing length for large and small mosquitoes was 3.4 ± 0.03 mm and 2.7 ± 0.04 mm, respectively (Fig. 1C). The average wet and dry body weight of large mosquitoes was 2.73 ± 0.06 mg and 1.21 ± 0.03 mg, respectively, whereas the small mosquitoes had an average wet and dry body weight of 1.28 ± 0.08 mg and 0.50 ± 0.04 mg, respectively (Fig. 1D, E). The average wing length of large female mosquitoes was significantly longer than the wing length of small female mosquitoes (unpaired *t*-test *t* = 15.11; degrees of freedom (*df*) = 98; *P* < 0.0001). Likewise, the wet and dry body weight of large mosquitoes was significantly higher than the weight of the small mosquitoes (unpaired *t*-test, *t* = 14.43; *df* = 98; *P* < 0.0001).

### Small mosquitoes are less active than large mosquitoes

**Fig. 2:**
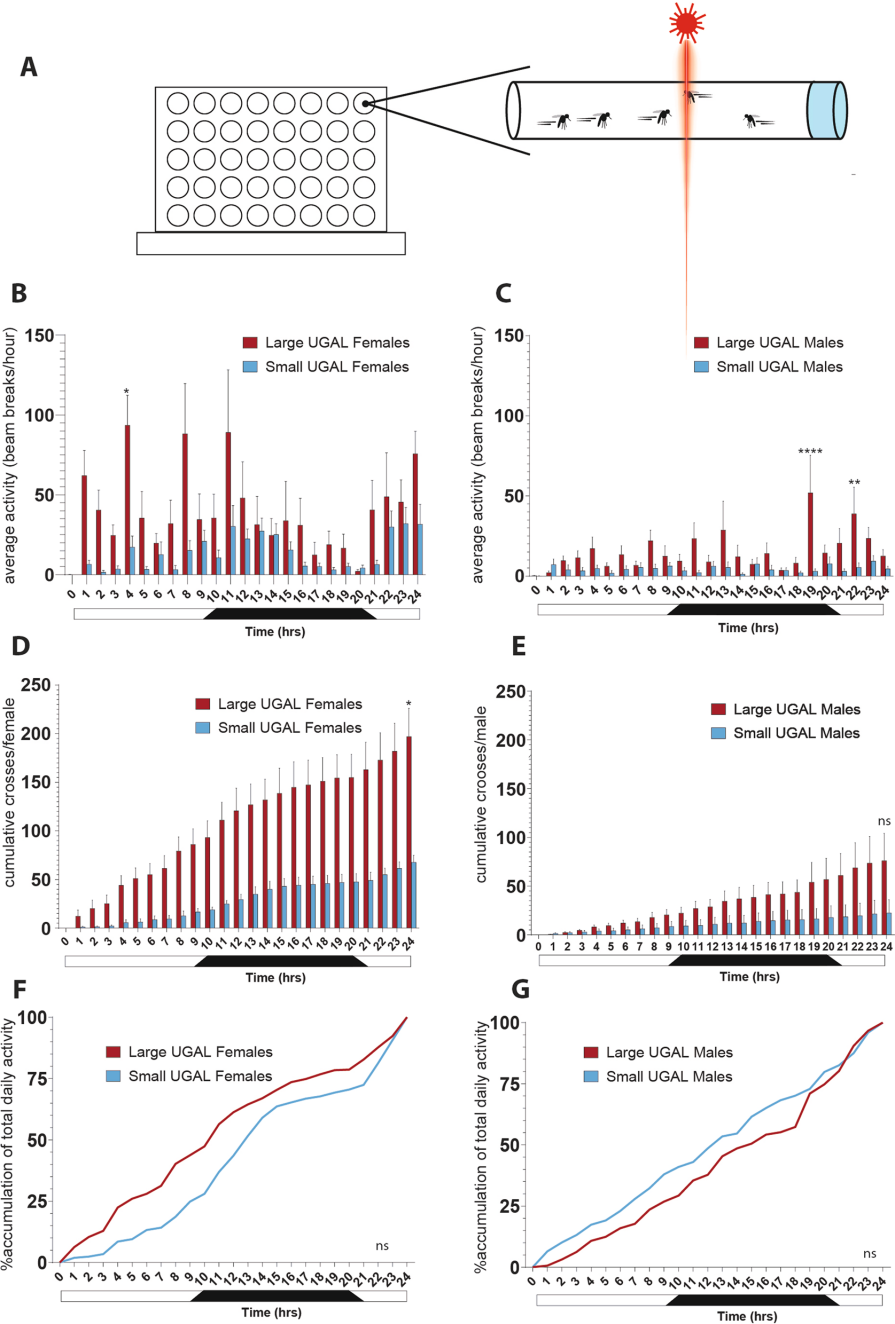
Locomotor activity of large and small mosquitoes over a 24-h time period. **A** Schematic representation of LAM setup. **B**, **C** Average locomotor activity during 60-min time periods. Mann–Whitney *U* test was performed. ^*^P < 0.05. **D** and **E** Cumulative average crosses per individual mosquitoes. Unpaired *t*-test was performed. ^*^P < 0.05. **F** and **G** Accumulation dynamics of locomotor activity over 24 h. Mann–Whitney *U* test was performed when 50%, 75%, and 95% of total daily activity occurred. ^*^P < 0.05. Light and dark hours are indicated by the white and black bar above each figure panel. Error bars represent the standard error of the mean (SEM)

We used the LAM-25 to record the locomotor data of the two groups of mosquitoes. Average crossings within 60 min were recorded to assess the activity patterns of mosquitoes over 24 h (Fig. 2A). Both male and female large mosquitoes exhibited more locomotor activity compared with their small counterparts (Fig. 2B, C). Mann–Whitney *U* test showed a statistically significant difference in 24-h cumulative crosses per individual mosquito between large and small females (Fig. 2D). Large females had median daily crosses of 191.1 ± 28.93 counts per day, while the small females had a median daily crosses of 69.43 ± 7.09 counts per day (*P* < 0.05). Male mosquitoes showed differences in 24-h cumulative crosses per individual mosquito, this was not significantly different (Fig. 2E). Large males had median daily crosses of 70.43 ± 27.98 counts per day, while the small males had median daily crosses of 12.33 ± 13.66. Large males had 58.10 counts more than small males, but there were no significant differences. The accumulation dynamics of locomotor activity over 24 h showed differences in the activity patterns between large and small groups with no significant differences (Fig. 2F, G).

In females, large mosquitoes reached 50% of their daily total activity by a median time of 10 h 50 min, whereas small males achieved this 3 h 50 min later, at a median time of 14 h. For males, the opposite was found; small males achieved 50% of their total daily activity by a median time of 13 h 50 min, whereas the large males achieved this 1 h 50 min later, at a median time of 15 h. The time when 75% of daily total flight activity was accomplished was similarly different between the large and small female mosquitoes, with large females reaching 75% of total daily activity at a median time of 16 h and small females at a median time of 21 h 50 min, 5 h 50 min later. Male mosquitoes again displayed the opposite trend; small males achieved 75% of their daily flight activity by a median time of 20 h, whereas the large males followed 1 h later, at a median time of 21 h. By the time of 95% accumulation in flight activity, no size-based difference was detected.

### Large and small mosquitoes display different feeding dynamics but take the same number of blood meals over 2 weeks

**Fig. 3:**
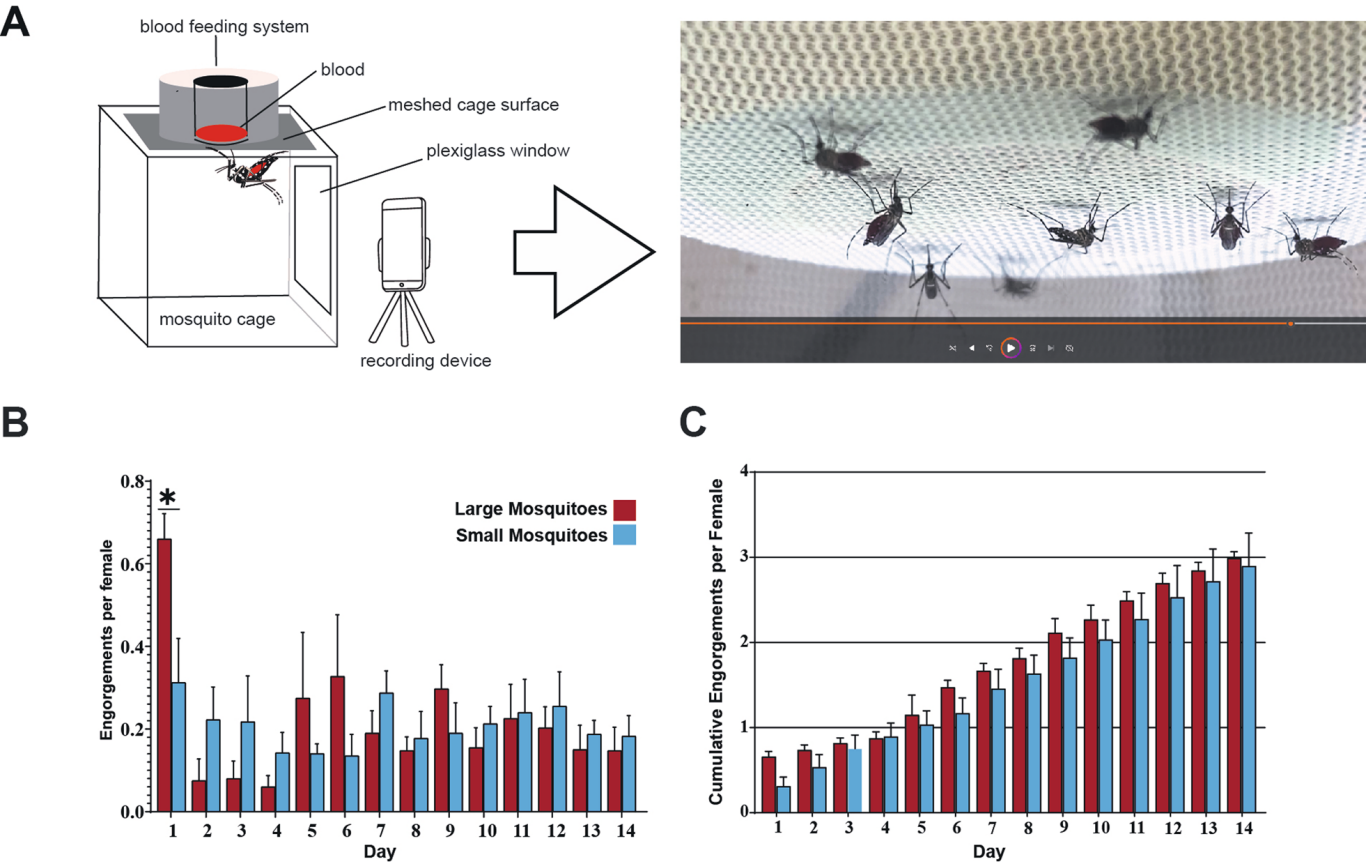
Feeding behavior of large and small *Aedes aegypti*. **A** Schematic of feeding frequency assay. **B** Average daily engorgement rates of four cages of large and small mosquitoes each, over 2 weeks. **C** Cumulative engorgements per female over 2 weeks. Error bar represents the standard error of the mean (SEM). Unpaired *t*-tests were performed for each day. ^*^P < 0.05

Large and small mosquitoes were offered blood meals for 30 min per day for 2 weeks under video surveillance (Fig. 3A). The number of engorging females for each day were recorded. We calculated average daily rates of imbibement per cage of mosquitoes (Fig. 3B). We found that small and large mosquitoes had different feeding patterns over the 2-week course. However, the rate of engorged females each day was not significantly different between groups, except for day 1 (Unpaired *t*-test, *t* = 2.82; *df* = 6; *P* < 0.05). We analyzed the total number of engorgements at the end of the 2 weeks and found that there were no significant differences in the total number of engorgements per female (Fig. 3C).

### Small female mosquitoes from a pesticide-resistant strain show more resistance to permethrin than large female from the same strain

**Fig. 4:**
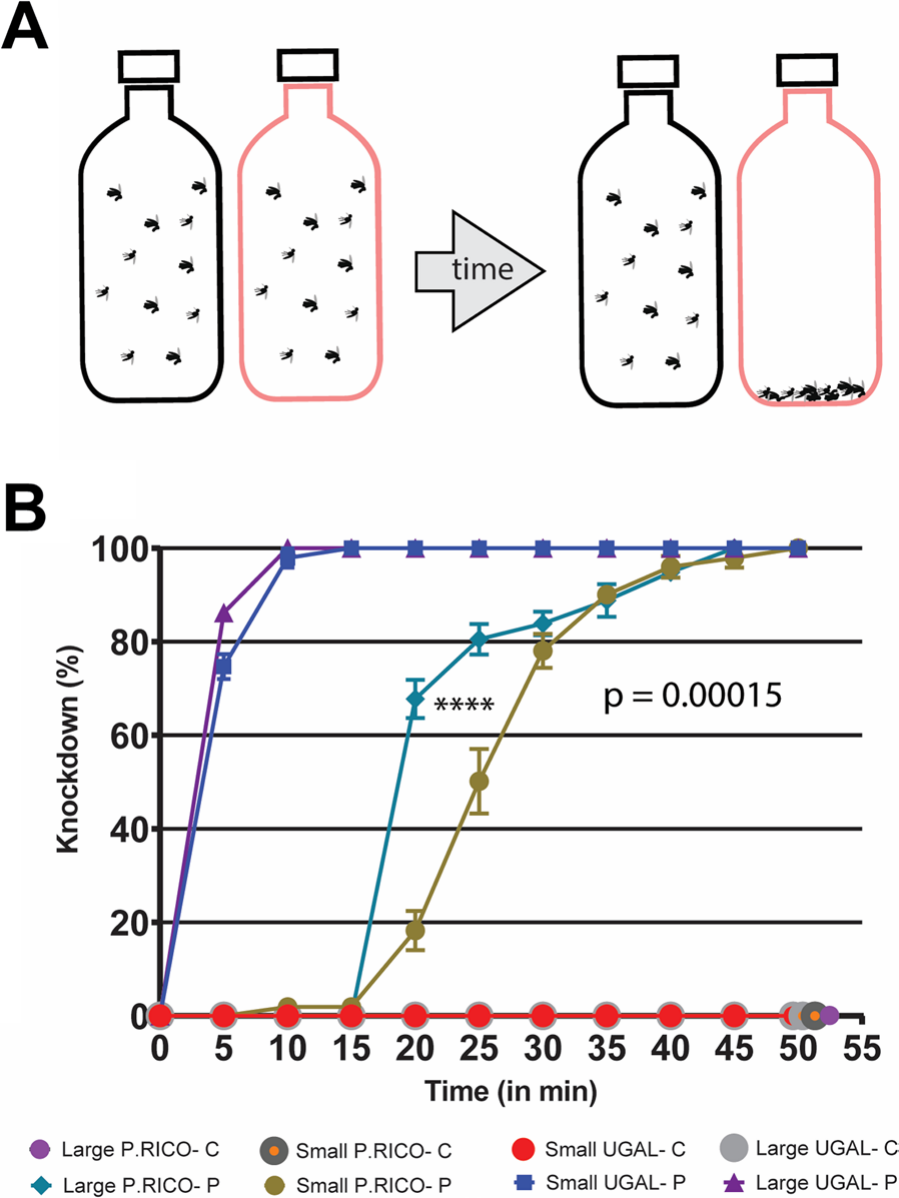
Insecticide resistance of large and small *Aedes aegypti*. **A** schematic of the bottle assay. The red-outlined bottle represents a permethrin-coated bottle. **B** Percent knockdown for small and large female mosquitoes of *Aedes aegypti* UGAL and Puerto Rico (P. Rico) strains exposed to control (Acetone) or 86 μg permethrin over 50 min. All data points are presented as average percent knockdown ± standard error of the mean (SEM). P. Rico, Puerto Rico; C, control; P, permethrin

We performed bottle assay tests to assess levels of insecticide resistance in small and large mosquitoes of different strains. The test result showed that both sizes of UGAL strain mosquitoes were extremely sensitive to permethrin and had high knockdown rates within the first 5 min (Fig. 4). Small and large UGAL knockdown rates were not significantly different across the time course of the assay (*P* > 0.05). Puerto Rico mosquitoes showed strong insecticide resistance, with knockdown curves shifted about 20 min to the right. Small Puerto Rico females did show significantly more resistance to permethrin than their larger counterparts (Fisher exact test, two-sided, *P* < 0.05).

## Discussion

Rising global temperatures are likely to expand the geographical range of important vector mosquito species, such as *Ae. aegypti *[20, 21]. Higher environmental temperatures have been shown to affect mosquito sizes [22, 23]. Changes in factors such as the quality of larval habitat, food availability, temperature, and inter- and intra-species competition result in large variations in adult mosquito body size in wild populations [24, 25]. In contrast, under controlled laboratory rearing conditions, mosquitoes are usually larger and more uniform in size than their wild counterparts [26, 27]. The effects of size variation on mosquito physiology and vectorial capacity are an understudied field in vector biology. The current study was designed to test the hypothesis that small mosquitoes have characteristic traits that increase their vectorial capacity compared with large mosquitoes. To investigate this, we compared small and large mosquitos’ locomotor activity, biting frequency, and insecticide resistance. We suggest that these three variables can be used, among others, as predictors for wild mosquito vectorial capacity.

### Small mosquitoes are half the size of large mosquitoes and display wing length–mass allometry

As expected from the literature, larval crowding and starvation resulted in small adult mosquitoes, with half the weight of the large control mosquitoes [28]. Interestingly, the wing length of small mosquitoes was only 20% shorter than the large mosquito wing lengths (Fig. 1). This type of hypoallometry in mosquitoes has been described in the literature [22, 29]. The consequences on flight performance caused by wing length–mass disproportion in mosquitoes have yet to be determined. Flight is not the only variable that may be impacted by this allometry. A study from 2021 conducted by Nadai and co-workers found that temperature and female mosquito body size influence wing-beat frequency [30]. Since wing-beat frequency and the ability to match the wing-beat frequency is important for male mosquitoes to locate females of their own species and successfully mate [31, 32], we speculate that changes in this frequency influences mating performance. This proposes the idea that flight and mating performance might be impacted by mosquito size and wing proportions. A study by Schneider and coworkers describes mosquito wing lengths in field-collected females from Iquitos, Peru [33]. They found that wing length varied significantly, with the largest mosquitoes slightly larger than our large mosquitoes (3.8 mm versus 3.4 mm) and the smallest significantly smaller than our small mosquitoes (1.7 mm versus 2.7 mm).

### Small mosquitoes are less active

*Ae. aegypti* mosquitoes can fly as far as a few hundred meters during their lifespan. Mosquitoes travel to locate hosts, mates, resting sites, and oviposition sites [34, 35]. Flight activity plays a crucial part in the swarming, mating, and dispersal of mosquitoes [32, 36]. It is well-understood that mosquitoes across species have specific diurnal activity cycles. During peaks of activity, mosquitoes are more likely to host-seek, bite, and thus transmit pathogens [37–39]. Using the Locomotor Activity Monitor (LAM), we compared general mosquito activity over one diurnal cycle in large and small mosquito groups (Fig. 2). As expected with *Ae. aegypti*, we observed diurnal changes in activity for both the large and small females with low activity during late night [40]. Interestingly, the male mosquito diurnal activity for both groups was less clear, and there was overall less activity was observed in the LAM. Small mosquitoes, of both sexes, were less active compared with their large counterparts. By the end of the 24-h testing period, large mosquitoes were approximately three times more active than small mosquitoes. A similar behavior has been observed in *Drosophila melanogaster*, where smaller flies were less active than larger flies [41, 42]. On the basis of these results, we predict that smaller mosquitoes have limited average dispersal ranges and daily movement compared with larger mosquitoes. To assess this hypothesis, we suggest the use of mosquito mark–release–recapture (MMRR) experiments [43, 44] or the use of individual-tracking equipment such as harmonic radar [45].

### Adult biting frequency was similar between small and large *Ae. aegypti* mosquitoes

Under optimal laboratory conditions, the typical gonotrophic cycle of *Ae. aegypti* mosquitoes from the ingestion of a blood meal to egg deposition takes 72 h. Interestingly, small mosquitoes required more than one blood meal to complete a single gonotrophic cycle, thus increasing the timeframe of this cycle. This is likely due to inadequate nutrient reserves from the aquatic stage of their life cycle. Shiao and collaborators showed that treating small mosquitoes with juvenile hormone before a blood meal eliminates the need for a second meal to lay eggs [46]. On the basis of this study, we hypothesized that small mosquitoes bite more frequently than large mosquitoes, hence making them more dangerous. We observed different patterns in biting behavior and gonotrophic cycle length between small and large mosquitoes over time. However, surprisingly the cumulative number of bites over 2 weeks was similar between both groups (Fig. 3). Therefore, our hypothesis was not supported by this experimental data.

### Small size increases insecticide resistance only in a pyrethroid-resistant strain of *Ae. aegypti*

Resistance to pyrethroids, a common class of insecticides, in *Ae. aegypti* is a worldwide phenomenon that poses significant challenges for vector control efforts [17, 19, 47–49]. The molecular mechanisms of pyrethroid-resistance are well understood [50–52]. However, to our knowledge, the impact of mosquito size on insecticide resistance has not been investigated. Our initial hypothesis was that small mosquitoes would be more susceptible to pyrethroids compared with large mosquitoes because they would uptake a relatively higher dosage in the bottle assay. Contrary to our hypothesis, we found that mosquito size did not impact the knockdown curve of our susceptible UGAL strain and that small mosquitoes from the Puerto Rico strain were more resistant than their large counterparts (Fig. 4). These findings are puzzling, and understanding the mechanism of size on pesticide resistance requires an in-depth investigation.

### Concluding notes: Are small mosquitoes more dangerous than large mosquitoes?

With rising global temperatures, the average size of mosquitoes may decrease in select regions [24, 53]. Here, we assessed whether mosquito size impacts variables related to their vectorial capacity: activity, biting, and insecticide resistance. We found that small mosquitoes are generally less active than large mosquitoes, that they bit the same number of times over a 2-week period, and that, in insecticide-resistant strains, small size was associated with decreased susceptibility to insecticides.

Mosquito feeding behavior is complex, and its study is prone to experimental bias [54]. A critical question is whether our results obtained under laboratory conditions also apply under field conditions. Studies by Scott and coworkers clearly show that *Ae. aegypti* females often bite multiple times during a single gonotropic cycle under both field and laboratory conditions [55, 56]. In our feeding assay setup, it is possible for a skip-feeding individual mosquito to be counted multiple times during the 1-h feeding intervals. In the field, skip-feeding mosquitoes may bite multiple hosts, and this behavior might differ in large and small mosquitoes. To detect this behavior, individual labels could be attached to mosquitoes. Accordingly, flight activity in the LAM may not represent flight activity in the field for large and small mosquitoes. Testing flight activity in the field is difficult but could be accomplished by release/recapture studies. Another question not addressed in our study is whether small and large mosquitoes have different host preferences and whether feeding behavior and patterns in cage experiments depend on the type of blood used in the study. The nutritional value of blood from different hosts (e.g., human versus bovine) can affect reproductive fitness in mosquitoes [54]. However, whether this results in variations in their behavioral patterns is unknown.

Variables that are missing from our study but should be investigated in the future to determine whether small mosquitoes are more dangerous than large ones are mating success, reproductive success, dispersal patterns and distances, and vector competence for specific pathogens.

## Conclusions

The findings of our study do not conclusively support the idea that mosquito size impacts vectorial capacity. Therefore, the question remains unanswered at this time.

## Supplementary Information

Supplementary Material 1: S1: Raw data from this study. This Excel spreadsheet contains the raw data and results of the statistical test that we performed for the individual figures

Supplementary Material 2: Movie clips from biting assay. S2 Large mosquitoes

Supplementary Material 3: Movie clips from biting assay. S3 Small mosquitoes

## Data Availability

The data that support the conclusions of this study are included within the Supplementary Information files.
